# The mitochondrial genome of *Huananpotamon koatenense* (Rathbun, 1904) (Brachyura, Potamidae) and phylogenetic analysis

**DOI:** 10.1080/23802359.2024.2391086

**Published:** 2024-08-13

**Authors:** Yunlong Sun, Bing Wang, Meijun Liu, Yifan Wang, Kelin Chu, Linna Lv

**Affiliations:** a College of Life Science, Nanjing Normal University, Nanjing, China; b Nanjing Institute of Environmental Sciences, Ministry of Ecology and Environmental of the People’s Republic of China, Nanjing, China

**Keywords:** Freshwater crab, China, mitogenome, Potamiscinae, Decapoda

## Abstract

Freshwater crabs play essential roles in the well-functioning of the inland aquatic ecosystems. However, due to the lack of sufficient molecular resources, the study of freshwater crabs has been greatly hindered. In this study, the mitochondrial genome of *Huananpotamon koatenense*, a freshwater crab endemic to China, was sequenced for the first time. This mitogenome sequence is 15,528 bp long, and contains 13 protein-coding genes, 2 rRNA genes and 22 tRNA genes. Phylogenetic analyses based on 25 mitogenomes showed that *H. koatenense* was clustered with the known congeneric species of *H. lichuanense*.

## Introduction

True freshwater crabs (hereafter, freshwater crabs) undergo direct development and complete their life cycle in freshwater or land (Yeo et al. 2008; Cumberlidge and Ng 2009). Because of their low dispersal ability and fecundity, freshwater crabs are usually considered excellent models for studying biogeography (Shi et al. 2021; Pan et al. 2022). However, due to the lack of molecular resources and the taxonomic ambiguity of many taxa, e.g. *Indochinamon* and *Potamiscus*, the study of freshwater crabs has been greatly hindered (Pan et al. 2022).

The freshwater crab genus *Huananpotamon* Dai & Ng, 1994 (Potamidae) is endemic to Wuyi Mountains in eastern China (Wang et al. 2022). With limited distribution range, *Huananpotamon* represents the third speciose Potamidae genus in China, which could potentially be the result of vicariant speciation. However, the lack of molecular resources has hindered the understanding of its evolutionary history. We here sequenced the mitochondrial genome of *Huananpotamon koatenense* (Rathbun, 1904) and performed phylogenetic analyses based on 13 PCGs. Our results could serve as molecular resources for further studies of freshwater crabs.

## Materials and methods

**Figure 1.**
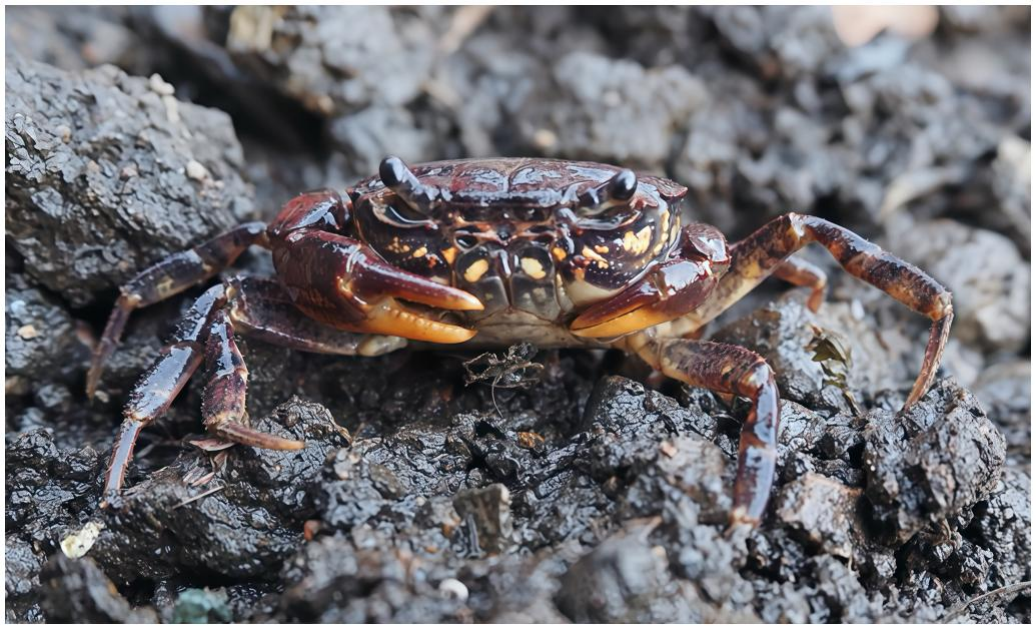
*Huananpotamon koatenense* from Wuyishan National Park, Fujian, China. This image was taken by Ruxiao Wang.

A live individual of *H. koatenense* (Figure 1) was collected in Tongmu Village, Wuyishan National Park, Fujian, China (27.7068°N, 117.6911°E), preserved in 95% ethanol at room temperature, and deposited in the Jiangsu Key Laboratory for Biodiversity and Biotechnology, College of Life Sciences, Nanjing Normal University, China (contact person and email: YLS sunyunlong1998@163.com, voucher number: NNU11761). The specimen can be identified as *H. koatenense* by a combination of traits, including carapace broader than long; postorbital cristae distinct, slightly rugose, confluent with epibranchial teeth; third maxilliped exopod with flagellum; and male first gonopod terminal segment expanded, inner-distal angle prominent, outer-distal angle elongated, dagger-shaped (Wang et al. 2022). Genomic DNA was extracted from gill tissue of the ethanol preserved specimen with the Cell and Tissue DNA Extraction Kit (Generay Biotech) following the manufacturer’s protocol.

A library was constructed using the TruSeq DNA PCR-free prep kit (Illumina) and sequenced on the Illumina Novaseq 6000 platform in Personalbio (Shanghai China). Information about sequencing details: Insert Size is 400 bp and Sequencing Mode is Paired-end 2 × 150bp. A total of 6,620,397 reads were obtained.

The mitogenome was assembled using MitoZ v2.4 (Meng et al. 2019) and NOVOPlasty v4.3.1 (Dierckxsens et al. 2017), annotated with MitoS2 WebServer (Bernt et al. 2013), and visualized with Proksee WebServer (Grant et al. 2023). The *cox1* gene sequence of *Huananpotamon lichuanense* (MN737141, Zhang et al. 2020) was used as a seed in NOVOPlasty. The resultant mitogenome sequence is linear and incomplete. We failed to recover the complete mitogenome through Sanger sequencing. The reads were mapped to the assembled sequence with BWA v0.7.17-r1188 (Li and Durbin 2009). Read depth at each position was calculated using SAMtools v1.7 (Li et al. 2009) and then plotted using python (Supplementary Figure S1). AT-rich regions show high read coverage (Supplementary Figure S1). Tandem repeats were identified using Tandem Repeats Finder v4.09 (Benson 1999).

Based on Pan et al. (2022), *Huananpotamon* was placed in the ‘Sout China-adjacent Islands clade’. Within this clade, all species with available mitogenome sequences in the NCBI database were selected as in-groups. In addition, representatives from the other clades were selected as out-groups. As a result, mitogenomes of *H. koatenense* and 24 other species of Potamidae were included to construct the phylogenetic tree (Table 1). Nucleotide sequences of 13 protein-coding genes (PCGs) were aligned using MAFFT v7.310 with the G-INS-i strategy (Katoh and Standley 2013) and concatenated using MEGA X (Kumar et al. 2018). The phylogenetic analyses were performed using the Maximum-likelihood (ML) method with IQ-TREE v2.2.0 (Minh et al. 2020). ML analyses clade support values were evaluated using 1000 ultrafast bootstrap replicate searches (Minh et al. 2013). To identify the best substitution model for the ML and Bayesian analyses, we employed ModelFinder (Kalyaanamoorthy et al. 2017) within IQ-TREE2. The TIM2 substitution model, with empirical base frequencies, a proportion of invariant sites, and discrete Gamma model (TIM2 + F + I + G), was selected as the best-fit model. Bayesian inference (BI) analyses were conducted with MrBayes v3.2.7 (Ronquist et al. 2012). Four independent runs were performed with 5 million generations and sampling every 1,000 generations. The average standard deviation of the split frequencies (ASDSF) was lower than 0.01. Convergence and the adequacy of the burn-in were further assessed with Tracer v.1.7.2 (Rambaut et al. 2018). The first 25% of MCMC chains were discarded as burn-in. In addition, all parameters had effective sampling sizes (ESS) greater than 200. Phylogenetic trees inferred by both methods were visualized and edited with iTOL (Letunic and Bork 2019).

**Table 1.** Species names, GenBank accession numbers and references of all sequences used to construct phylogenetic trees.

Species	Accession number	Reference	
*Apotamonautes hainanensis*	MN737137	Zhang et al. 2020	
*Bottapotamon chenzhouense*	OR687237	Unpublished	
*Bottapotamon lingchuanense*	MN117717	Wang et al. 2021b	
*Bottapotamon engelhardti*	OR687236	Unpublished	
*Bottapotamon fukienense*	OR699219	Unpublished	
*Bottapotamon nanan*	OR699220	Unpublished	
*Bottapotamon yonganense*	OR699221	Unpublished	
*Bottapotamon youxiense*	OR687238	Unpublished	
*Candidiopotamon okinawense*	MN737145	Zhang et al. 2020	
*Chinapotamon depressum*	MZ350918	Pan et al. 2022	
*Chinapotamon maolanense*	MT134100	Cui et al. 2020	
*Geothelphusa albogilva*	MZ350921	Pan et al. 2022	
*Geothelphusa dehaani*	AB187570	Segawa and Aotsuka 2005	
*Hainanpotamon daiae*	MZ350922	Pan et al. 2022	
*Huananpotamon koatenense*	OQ091257	Present study	
*Huananpotamon lichuanense*	KX639824	Bai et al. 2018	
*Indochinamon daweishanense*	MZ350926	Pan et al. 2022	
*Nanhaipotamon hongkongense*	MW125541	Wang et al. 2021a	
*Nanhaipotamon pinghense*	MZ350931	Pan et al. 2022	
*Neotiwaripotamon jianfengense*	MZ350933	Pan et al. 2022	
*Neotiwaripotamon whiteheadi*	MZ350934	Pan et al. 2022	
*Sinolapotamon cirratum*	OR687241	Unpublished	
*Sinolapotamon palmatum*	OR687242	Unpublished	
*Sinolapotamon patellifer*	MK883709	Ji et al. 2019	
*Tenuipotamon panxiense*	MZ350954	Pan et al. 2022	

## Results

**Figure 2.**
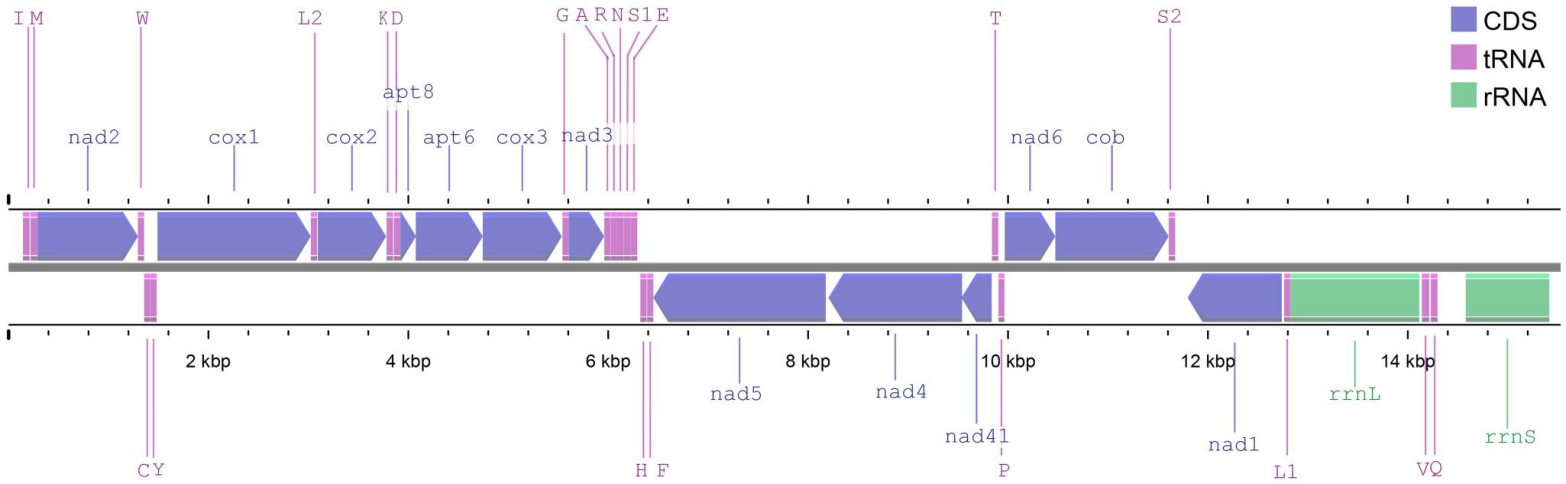
Linear map of the sequenced part of *Huananpotamon koatenense* mitogenome, with a total length of 15,528 bp. There is no indication that the complete mitogenome is actually linear.

The sequenced part of *H. koatenense* mitogenome has a length of 15,528 bp, and encodes 37 mitochondrial genes, 13 protein-coding genes (PCGs), 22 transfer RNA (tRNAs) genes, and two ribosomal RNA (rRNAs) genes (Figure 2). The non-coding region locates between *rrnS* and *trnI*. The overall nucleotide composition of the sequenced part of the mitogenome is *A* = 35.9%, *T* = 37.7%, *G* = 9.2%, and *C* = 17.2%, with a GC content of 26.4%. The initiator codons of all 13 PCGs are ATN. All PCGs stop with TAA except for *atp8* which stops with TAG and *cob* which stops with an incomplete stop codon T. The lengths of the PCGs vary from 159 bp (*atp8*) to 1,659 bp (*nad5*), collectively constituting 70.9% of the sequenced part of the mitogenome sequence. The A + T content of all 13 PCGs is 71.9%. Three tandem repeats are identified. The longest motif is 20 bp with 2.4 repeats. The other two motifs are 17 bp with 1.9 repeats and 16 bp with 2.3 repeats.

**Figure 3.**
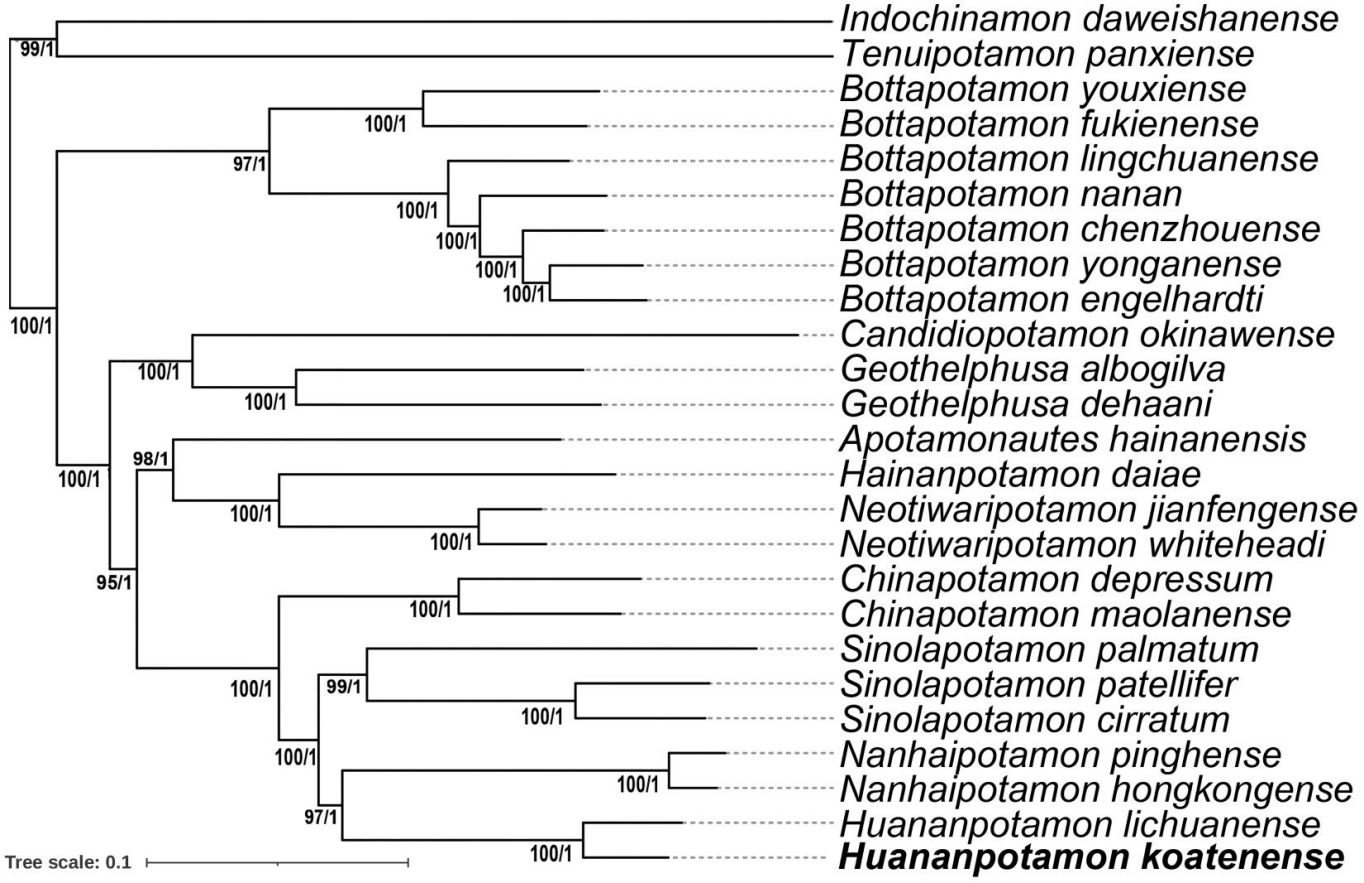
Maximum-likelihood (ML) phylogenetic tree based on the nucleotide sequence of 13 PCGs. Numbers on the branches indicate bootstrap values and posterior probabilities. Scale bar represents the expected number of substitutions per site. The newly sequenced mitogenome is shown in bold font. The reference data for sequences used in phylogenetic reconstructions are presented in Table 1.

Phylogenetic trees inferred by ML and Bayesian methods yielded the same topology and largely strong node support (Figure 3). Phylogenetic analyses showed that *H. koatenense* was clustered with the known congeneric species of *H. lichuanense* (Figure 3).

## Discussion and conclusion

This study determines the first mitochondrial genome of *H. koatenense*. The gene order of the *H. koatenense* mitogenome is same as previously studied species of the genus *Huananpotamon* (Figure 2), which putatively represents the ancestral state of the gene order in the subfamily Potamiscinae (Zhang et al. 2020). The A + T content of all 13 PCGs is similar to that of other potamiscine crabs (Zhang et al. 2020; Pan et al. 2022) but slightly higher than that of gecarcinucid crabs (Du et al. 2022). The mitogenome of *H. koatenense* reported in this study will contribute to further research on the taxonomy and systematics of *Huananpotamon*, deepening our understanding of the evolutionary history of this speciose genus.

## Supplementary Material

Supplemental material.pdf

## Data Availability

The data supporting this study’s findings is openly available in GenBank of NCBI at https://www.ncbi.nlm.nih.gov, reference number OQ091257. The associated BioProject, SRA, and Bio-Sample numbers are PRJNA936093, SRR23517942, and SAMN33336541 respectively.
