# Supplementary material for: The mitochondrial genome of Huananpotamon koatenense (Rathbun, 1904) (Brachyura, Potamidae) and phylogenetic analysis
Source: Mitochondrial DNA B Resour. 2024 Aug 13;9(8):1068–71. doi: 10.1080/23802359.2024.2391086 (PMC11328792; doi:10.1080/23802359.2024.2391086)
Supplement: Supplemental material.pdf [file TMDN_A_2391086_SM0807.pdf]

Supplemental material

The mitochondrial genome of *Huananpotamon koatenense* (Rathbun, 1904) (Brachyura, Potamidae) and phylogenetic analysis

Yunlong Sun[1], Bing Wang[1], Meijun Liu[1], Yifan Wang[1], Kelin Chu[2*], Linna Lv[1*]

[1] College of Life Science, Nanjing Normal University, Nanjing, China
[2] Nanjing Institute of Environmental Sciences, Ministry of Ecology and Environmental of the People's Republic of China, Nanjing, China

[*]**Corresponding authors:** llnlln1207@163.com; bsychukelin@163.com.

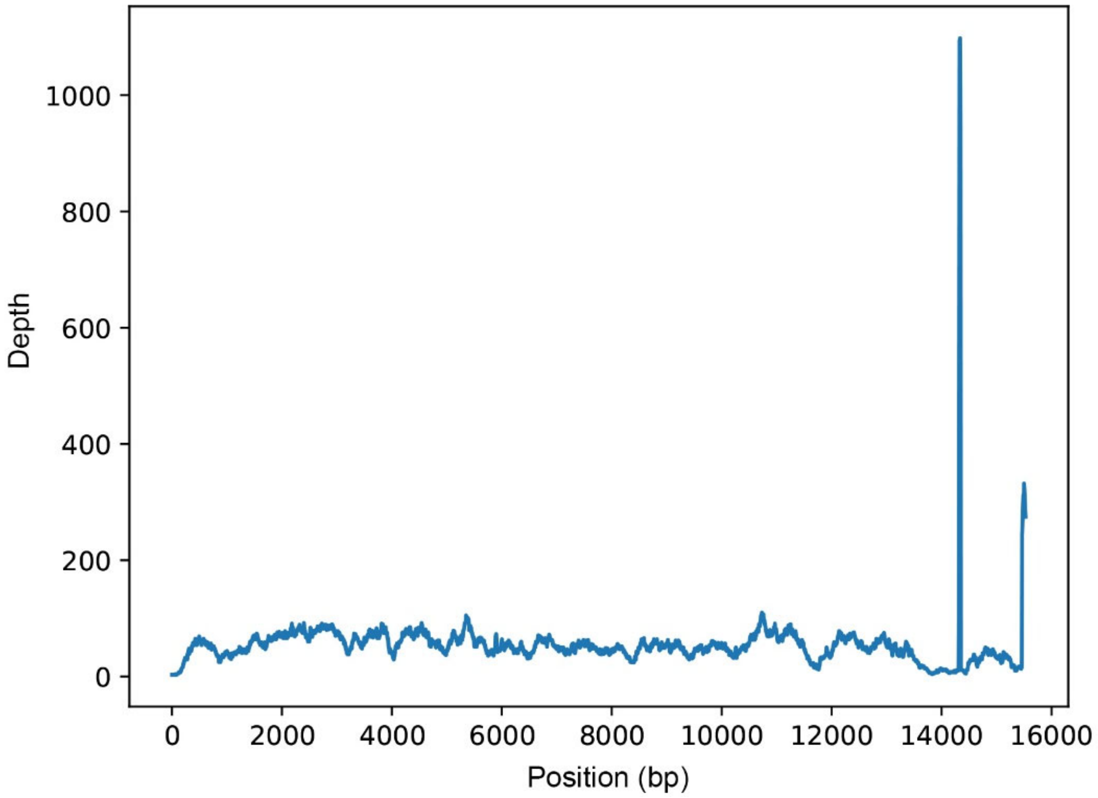

Supplementary Figure S1 Plot of the depth of coverage for short-read in the sequenced part of *Huananpotamon koatenense* mitogenome (GenBank accession no. OQ091257). The horizontal axis represents the nucleotide position, and the vertical axis represents the read mapping depth.
